# Simultaneous Measurement of Transcriptional and Post-transcriptional Parameters by 3′ End RNA-Seq

**DOI:** 10.1016/j.celrep.2018.07.104

**Published:** 2018-08-28

**Authors:** Manfred Schmid, Agnieszka Tudek, Torben Heick Jensen

**Affiliations:** 1 Department of Molecular Biology and Genetics, Aarhus University, C. F. Møllers Allé 3, Bldg. 1130, 8000 Aarhus C, Denmark

**Keywords:** transcription, metabolic labeling, RNA-seq, RNA 3′, ends, poly(A)^+^, poly(A)^−^

## Abstract

Cellular RNA levels are determined by transcription and decay rates, which are fundamental in understanding gene expression regulation. Measurement of these two parameters is usually performed independently, complicating analysis as well as introducing methodological biases and batch effects that hamper direct comparison. Here, we present a simple approach of concurrent sequencing of *S. cerevisiae* poly(A)^+^ and poly(A)^−^ RNA 3′ ends to simultaneously estimate total RNA levels, transcription, and decay rates from the same RNA sample. The transcription data generated correlate well with reported estimates and also reveal local RNA polymerase stalling and termination sites with high precision. Although the method by design uses brief metabolic labeling of newly synthesized RNA with 4-thiouracil, the results demonstrate that transcription estimates can also be gained from unlabeled RNA samples. These findings underscore the potential of the approach, which should be generally applicable to study a range of biological questions in diverse organisms.

## Highlights

• Method using RNA 3′ end sequencing of total and 4tU labeled RNA
• pA^−^ RNA of labeled and total RNA mark nascent transcript ends at high resolution
• Dataset from single RNA samples can be used to measure transcription and RNA decay

## Graphical Abstract

**Figure:**
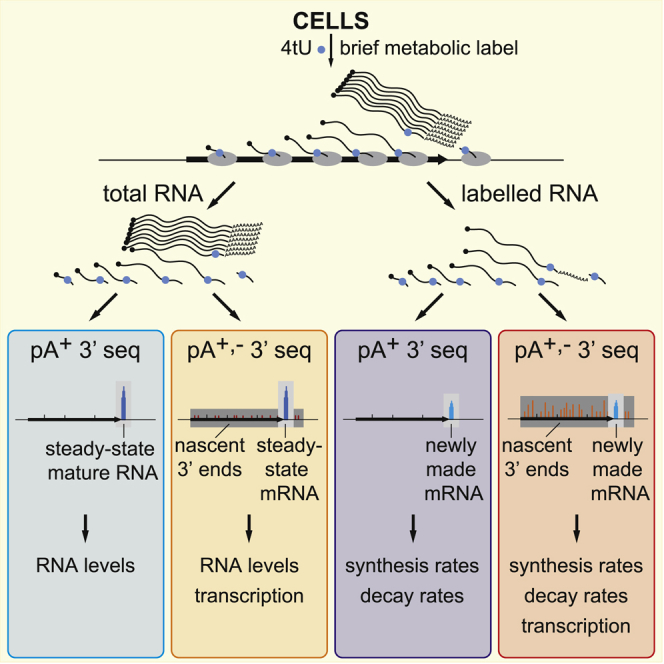

## Teaser

Schmid et al. present a method to interrogate transcription, total RNA levels, and RNA decay rates using pA^+^ and pA^−^ RNA 3′ end seq datasets from single RNA samples. pA^−^ RNA 3′ ends from 4tU labeled and total RNA mark nascent transcript 3′ ends at high resolution.

## Introduction

RNA transcription and decay determine cellular RNA levels, and their changes are important contributors to gene expression responses during cellular transition. Here, we use *S. cerevisiae* to establish a genome-wide method to simultaneously monitor these parameters from the same experimental sample.

Eukaryotic transcriptomes are complex, requiring consideration about their composition when applying techniques assessing transcription and turnover rates. The *S. cerevisiae* transcriptome consists of stable non-coding RNAs produced by RNA polymerase (RNAP) I and III, most commonly rRNAs and tRNAs, as well as a variety of coding and non-coding transcripts produced by RNAPII. The RNAPII-derived non-coding RNAs are diverse, but among the best-characterized classes are the small nuclear and nucleolar RNAs (snRNAs and snoRNAs), the cryptic unstable transcripts (CUTs), and the stable unannotated transcripts (SUTs) (Jensen et al., 2013, Wery et al., 2016, Wyers et al., 2005, Xu et al., 2009). While all newly synthesized RNAPII transcripts carry a m^7^G 5′cap, only protein-coding mRNAs and SUTs contain canonical 3′ end poly(A) (pA) tails produced by the pA polymerase Pap1p (Jensen et al., 2013, Xu et al., 2009). CUTs, on the other hand, are highly unstable and may transiently receive short oligo(A) tails by the TRf-Air-Mtr4 polyadenylation (TRAMP) complex, which facilitates their 3′–5′ decay. The stable snRNAs and snoRNAs are typically untailed, even though oligo(A) tailing by TRAMP may occur during their processing or quality control (Jensen et al., 2013, Roy et al., 2016, Wyers et al., 2005). TRAMP is targeted to these transcripts by the RNA-binding Nrd1p-Nab3p-Sen1p (NNS) complex, which also promotes RNAPII transcription termination (Porrua and Libri, 2015). Owing to the dense organization of the *S. cerevisiae* genome, many of the mentioned transcription units (TUs) are in immediate proximity and frequently overlap on opposite strands. Analysis of their transcription and RNA turnover therefore relies critically on strand-specific high-resolution methodologies. In fact, this consideration applies to most biological systems given the generality of pervasive transcription (Jensen et al., 2013).

Among contemporary techniques used to measure transcription activity, RNAPII chromatin immunoprecipitation (ChIP) is possibly the most broadly applied. ChIP allows for small-scale gene-specific analysis and genome-wide interrogation. In addition, a higher resolution variant termed ChIP-exo is available (Rhee and Pugh, 2011). However, the method comes with caveats: it lacks single-nucleotide resolution (which can partly be resolved by ChIP-exo), and it does not distinguish the transcribed from the non-transcribed strand. These limitations have prompted the development of alternative high-throughput techniques, which all provide both high resolution and strand-specific information. One of these is the global nuclear run-on sequencing (GRO-seq) technique and its successor, precision nuclear run-on sequencing (PRO-seq), which are elaborations of classical nuclear run-on analysis but now rely on metabolic, rather than radioactive, labeling of nascent RNA (Core et al., 2008, Kwak et al., 2013). While these approaches allow for excellent resolution and provide information on stranded-ness, they do not measure transcription rates in live cells but require the prior permeabilization of cells or purification of nuclei.

A panel of more recently developed (and related) approaches analyzes the RNA constituent co-purified with RNAP and residing in the active site of the enzyme. Among these, native elongating transcript sequencing (NET-seq), developed by Churchman and Weissman (2011), relies on purification of native RNAPII. Other laboratories have implemented technologies, where the nascent RNA is first cross-linked to RNAP before purification of the latter and analysis of the associating RNA (so-called RNAP cross-linking immunoprecipitation [CLiP] and cross-linking and analysis of CDNAs [CRAC]; Creamer et al., 2011, Milligan et al., 2016). This in principle permits the pinpointing of the exact position of RNAP. Lastly, another frequently employed approach estimates transcription rates by measuring chromatin-bound RNA as a proxy for nascent transcript synthesis (Carrillo Oesterreich et al., 2010, Mayer et al., 2015). In this case, it is not possible to readily distinguish RNA directly engaged with RNAPII from chromatin-bound post-transcriptional RNAs.

While ChIP sequencing (ChIP-seq), GRO-seq, NET-seq, and the CLiP/CRAC-seq variants are all considered state-of-the-art methods, allowing the measuring of transcription in a variety of settings, they all rely on advanced methodologies and expertise. Instead, a conceptually different approach is to measure RNA production in a defined time window using the metabolic labeling of live cells with 4-thiouracil (4tU), 4-thiouridine (4sU) or 5-bromouridine (5BrU) nucleotide analogs that incorporate into the nascent RNA chain. Combined with a measurement of total RNA levels, this allows for an estimate of RNA synthesis and decay rates from the same experiment and therefore provides a complete picture of gene expression (Miller et al., 2011, Neymotin et al., 2014, Rabani et al., 2014, Riising et al., 2014, Schwalb et al., 2016). However, in these protocols, it is not possible to distinguish transcriptional from post-transcriptional RNA. Protocol variants with brief labeling times combined with subsequent RNA fragmentation, before the purification of labeled RNA, partly resolve this by biasing toward measuring transcription (Riising et al., 2014, Schwalb et al., 2016). Still, even with brief (e.g., 5 min) labeling periods, the produced data contain a significant amount of post-transcriptional RNA (Schwalb et al., 2016). Thus, potential biases introduced by early post-transcriptional decay are disregarded, which is especially critical for the analysis of transcripts with short half-lives; i.e., RNAs subjected to nuclear turnover, underestimating transcription rates.

Here, we employ a 4tU-based method coupled to the direct sequencing of RNA 3′ ends to overcome these shortcomings. Exact positioning and pA-tail status determination of RNA 3′ ends allow us to interrogate transcription rates, RNA synthesis rates, and RNA half-lives independently and at high resolution from single RNA samples.

## Results

### Method Design

**Figure 1:**
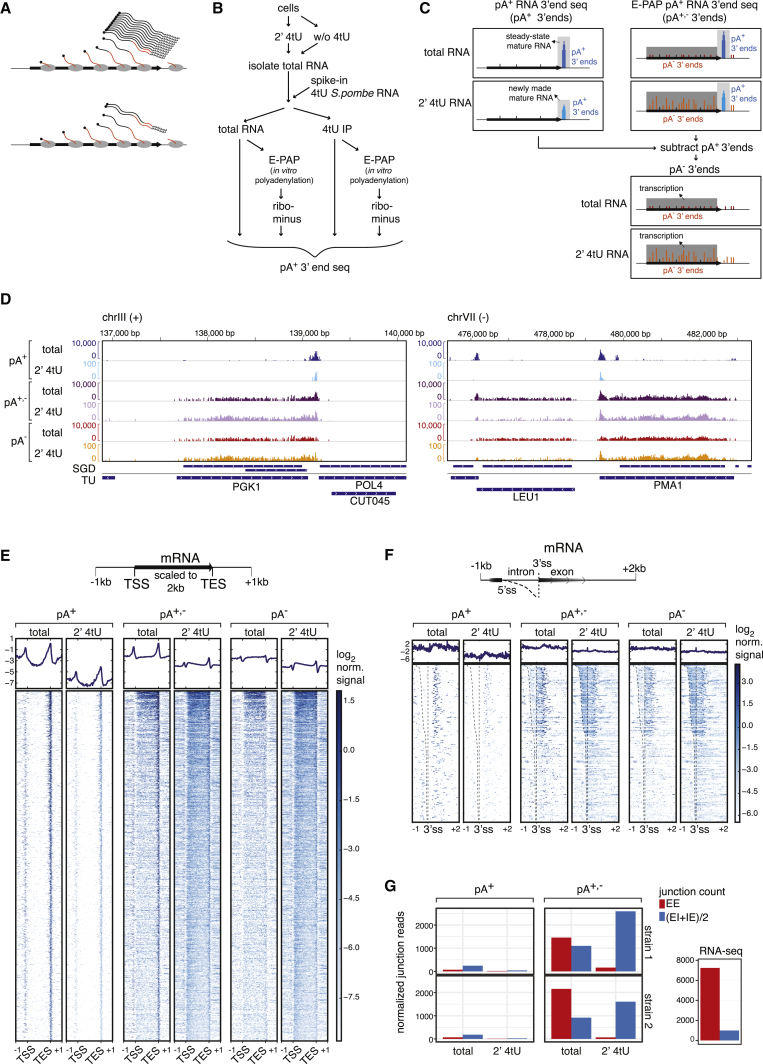
Genomic Position Analysis of pA^+^ and pA^−^ RNA 3′ Ends (A) Schematic representation of non-labeled (black) and metabolically labeled (red) fractions of total (top) or purified labeled (bottom) RNA. (B) Experimental strategy as outlined in the main text. (C) Workflow to derive pA^−^ 3′ ends. Theoretical distribution of RNA 3′ ends in the 2′ 4tU and total samples are depicted as explained in the main text. (D) Genome browser views of RNA 3′ ends around the *PGK1* (left) and *LEU1*-*PMA1* (right) loci. pA^+^ (top), pA^+,−^ (middle), and pA^−^ (bottom) log-scaled signals are shown from total and 2′ 4tU samples as indicated. Chromosomal coordinates and the strand for which data are shown are indicated on top. Annotations below each view region are from the *Saccharomyces* Genome Database (SGD; top), marking coding regions or mature isoforms of ncRNAs and transcribed regions according to Xu et al. (2009) (TU; bottom). (E) RNA 3′ ends as in (D) but shown as metagene profiles (top panels) and heatmaps (bottom panels). Log_2_ values are shown between all mRNA (n = 5,170) TSSs and TESs rescaled to 2 kb with 1 kb of non-scaled upstream and downstream regions as schematized on top. Metagene profiles display the mean of log_2_ values at each position. Rows of the heatmap are sorted by descending signal of the 2′ 4tU pA^−^ sample. (F) Metagene profiles (top) and heatmaps (bottom) as in (E) but around introns of protein-coding genes (n = 282). Data were aligned to intron 3′ splice sites (3′ss), including regions of 1 kb and 2 kb up- and downstream, respectively (as schematized on top), without scaling to length. Regions were ordered by descending intron sizes and dashed lines indicating 5′ss and 3′ss. (G) Quantification of exon-exon (EE), splice junction (exon-intron [EI] + intron-exon [IE]/2) read counts from pA^+^ and pA^+,−^ samples as well as total and 2′ 4tU libraries as indicated for the two yeast strains (left panels). A similar quantification of regular RNA-seq data from a control *S. cerevisiae* strain is shown on the bottom right. See also Figure S1 and Table S1.

Given the limitation of available 4tU-based protocols to discriminate transcriptional from post-transcriptional RNA, we reasoned that direct inspection of the 3′ end position and pA tail status of purified RNAs should provide such information. That is, their positioning away from annotated RNA 3′ ends and their lack of non-templated pA tails would suggest an origin within the catalytic center of RNAP and distinguish them from 3′ ends of mature transcripts positioned at annotated and/or experimentally determined gene 3′ ends. In the case of mRNAs and SUTs, this is the site of the pA tail (Figure 1A).

To pursue this idea, we developed an experimental strategy to map and quantify pA^+^ and pA^−^ 3′ ends from the same sample of total and newly synthesized *S. cerevisiae* RNA (Figure 1B). In brief, cells were incubated for 2 min with 4tU (2′ 4tU) and subjected to snap freezing and total RNA extraction. 4tU-containing RNA was then isolated and analyzed in parallel with an aliquot of the corresponding total RNA (total). In such experiments, the 4tU-labeling time constitutes a compromise between the need to be brief enough to enrich for nascent RNA while at the same time allowing for sufficiently pure 4tU-RNA samples. A labeling time of 2 min was chosen based on our preliminary experiments (Barrass et al., 2015; data not shown). To facilitate comparison between samples, a 1/100 w/w aliquot of total RNA from 4tU-labeled *S. pombe* cells was added as a spike-in. Finally, an additional sample was prepared from cells that were not labeled with 4tU to assess, and correct for, any unspecific background of the 4tU immunoprecipitation (IP). An aliquot of all RNA samples was then subjected to pA^+^ RNA 3′ end sequencing (pA^+^ RNA 3′ end seq) based on a commercially available dT-primed reverse transcriptase (RT) reaction (STAR Methods; Figure 1C, left). In parallel, a second aliquot was 3′end polyadenylated using *E. coli* pA polymerase (E-PAP), rRNA depleted, and subjected to pA^+^ RNA 3′ end sequencing, detecting both pA^+^ and pA^−^ 3′ends (pA^+,−^; Figure 1C, right top). Post-sequencing, positions of exact RNA 3′ ends were determined and quantified using the scheme outlined in Figure S1A. To specifically analyze pA^−^ 3′ ends as a potential transcription measure, pA^+^ reads were subtracted from pA^+,−^ reads (Figure 1C, bottom), aided by the use of *S.-pombe*-normalized read counts to ensure appropriate scaling. As expected, pA^+^ 3′ end signals were derived mostly from protein-coding genes, whereas pA^−^ signals were also derived from non-coding genes, which contributed the majority of reads in total RNA fractions (Figure S1B). Hence, using total RNA for analysis of pA^−^ signals from protein-coding and unstable non-coding TUs comes with the cost of lowered signal depth (see below). Even so, the results presented here could be achieved despite the low sequencing depth of 3–5 million reads per library (see Table S1).

### pA^−^ 3′ Ends Are Abundant Inside Gene Bodies

To evaluate the utility of the approach, we first scrutinized RNA 3′ ends derived from total and 2′ 4tU RNA preparations around the *PGK1*, *PMA1*, and *LEU1* genes (Figure 1D). As expected, pA^+^ 3′end library reads (pA^+^) originated almost exclusively from annotated transcript end sites (TESs) (Figure 1D, top tracks). In addition to these signals, the E-PAP-treated libraries (pA^+,−^) detected abundant and continuous RNA 3′ends within the respective gene bodies (Figure 1D, middle tracks). Finally, the subtraction-derived pA^−^ 3′ends were equally distributed over gene bodies and ends (Figure 1D, bottom tracks). These representations were generally valid for mRNAs (Figure 1E). The somewhat unexpected accumulation of pA^+^ 3′ ends close to the transcription start sites (TSSs) was due to the presence of pA^+^ 3′ ends of tandem upstream genes in the compact *S. cerevisiae* genome (Figure S1C). More importantly, total RNA also contained abundant internal pA^−^ 3′ ends, although the coverage and the relative levels were as expected lesser than in the 4tU IP samples (Figures 1D and 1E). Thus, despite the lower signals due to the dominating signal from non-coding pA^−^ ends in the total RNA noted above (Figure S1B), these samples also appeared amenable to the proposed strategy.

To assess whether internal pA^−^ reads represent genuine nascent RNA 3′ ends, we compared intronic and exonic signals from intron-containing protein-coding genes. In these cases, pA^−^ 3′ ends were present at roughly equal densities in introns and their downstream exons in the 2′ 4tU sample, whereas intronic reads were slightly depleted in the total sample (Figures 1F and S1D). The data were overall similar to published NET-seq, RNAPII CRAC, and RNAPII ChIP-seq datasets (Churchman and Weissman, 2011, Milligan et al., 2016, Warfield et al., 2017) but clearly distinct from total RNA-sequencing (RNA-seq) data where intronic signals are depleted (Figures S1D and S1E). Counting exon-exon (EE) and unspliced junction (exon-intron [EI] and intron-exon [IE]) reads supported these findings; while only few EI and IE junction reads were observed in the pA^+^ libraries, they were apparent in the E-PAP-treated libraries (Figure 1G, left [compare pA^+^ to pA^+,−^]). Moreover, EI and IE junction reads were dominating in the 2′ 4tU sample, whereas EE junctions were more prominent in the total sample (Figure 1G). Taken together, this indicated that pA^−^ 3′ ends from 2′ 4tU samples are derived from nascent transcripts, whereas the increased proportion of total RNA EE junction reads implied a contribution from 3′ to 5′ decay intermediates of spliced cytoplasmic mRNA. However, since the total sample contained a similar number of spliced and unspliced reads, even this library was biased toward non-spliced, presumably transcription-derived reads when compared to regular RNA-seq data, where EE junctions are over 5-fold more abundant than unspliced junction reads (Figure 1G, bottom right panel).

If pA^−^ 3′ ends are indicative of transcription, then their presence should extend downstream of pA sites. Indeed, at the level of single genes, pA^−^ signals were often detected beyond gene TESs as defined by pA^+^ signals and an ensuing drop in RNA-seq signals (Figure S1F). NET-seq and RNAPII CRAC data exhibited a similar pattern, indicating that pA^−^ 3′end signals downstream of TESs mark similar transcription intermediates. Considering all the data together, we therefore conclude that pA^−^ 3′ends are abundantly present inside of gene bodies and that both total and labeled RNA pA^−^ data appear useful for interrogating transcription.

### pA^−^ 3′ End Signals Provide Valid Transcription Measures

**Figure 2:**
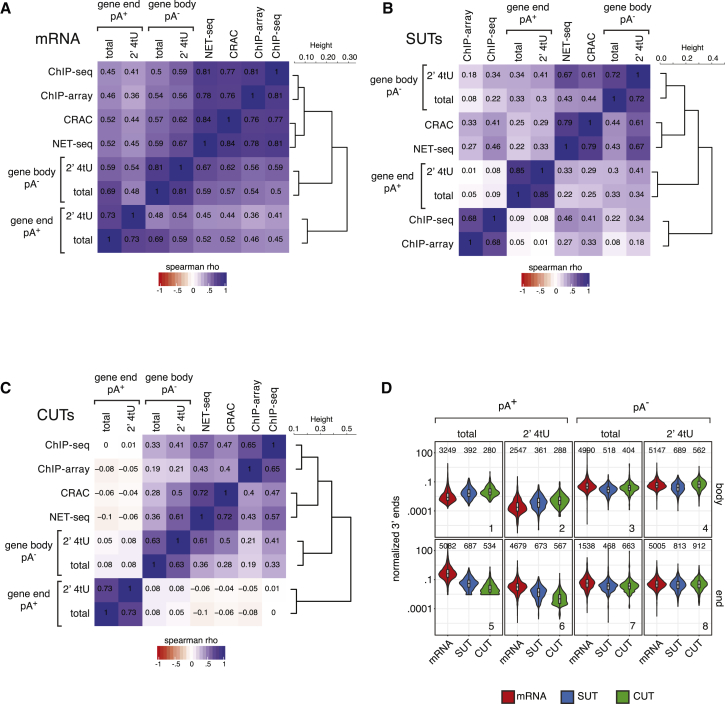
pA^−^ RNA 3′ End Counts Correlate with Other Transcription Measures (A) Spearman rank correlation (rho) matrix and hierarchical clustering of pA^−^ signal densities in gene body (TSS to 200 bp upstream of TES) and pA^+^ signals in gene end (TES ± 200 bp) regions of mRNAs (n = 5,171) and those reported from RNAPII ChIP-seq (Warfield et al., 2017), RNAPII ChIP-tiling array (ChIP-array; Mayer et al., 2010), NET-seq (Churchman and Weissman, 2011), and RNAPII CRAC (Milligan et al., 2016) datasets. (B) As in (A), but for SUT TUs (n = 847). (C) As in (A), but for CUT TUs (n = 925). (D) Violin plots depicting the distribution of signal densities in gene body and end regions for pA^+^ (left) and pA^−^ (right) signals. Values for mRNA, SUT, and CUT TUs are shown separately for total and 2′ 4tU samples. Number of TUs considered are depicted above each violin. See also Figure S2.

We next quantified pA^+^ and pA^−^ signals falling into gene body regions (from annotated TSSs to 200 bp upstream of annotated TESs) and gene 3′ end regions (the annotated TESs ± 200 bp), which are proposed to reflect transcriptional and post-transcriptional RNAs, respectively. Comparing gene body values to those of the above-mentioned published transcription estimates for mRNA (Figure 2A), SUT (Figure 2B), and CUT (Figure 2C) TUs yielded positive correlations, which were all statistically highly significant (Figures S2A–S2C), and highlighted the general reproducibility of the datasets throughout single replicates (Figure S2D). For mRNA TUs, there was also a highly significant correlation between gene 3′ end region pA^+^ signals and the reported transcription measures. This was expected for the 2′ 4tU RNA sample and for total RNA it probably reflects that mature mRNAs from highly transcribed genes are generally more abundant, because the transcription rate is the most important determinant of mRNA levels. Still, the correlation was weaker than considering pA^−^ 3′ends from gene bodies, underscoring the contribution of post-transcriptional decay in shaping the protein-coding transcriptome. Interestingly, like pA^−^ reads of 2′ 4tU RNAs, gene body pA^−^ reads from the total RNA samples also correlated well with transcription measures from unrelated techniques; this was particularly evident for mRNA and CUT TUs (Figures 2A, 2C, S2A, and S2C). This implies that the low amount of pA^−^ 3′ ends present in total RNA samples still contain a significant fraction of genuine nascent RNA 3′ ends. Instead, pA^+^ 3′ ends from the same sample were not, or only marginally, correlated with transcription levels for SUT and CUT TUs, consistent with their labile nature. We conclude that pA^−^ 3′ end signals inside genes can be used as reliable transcription measures, which is particularly clear for 2′ 4tU but also valid for total RNA.

We also analyzed the relative distributions of pA^+^ versus pA^−^ signals for the above-examined TUs. Consistent with mRNAs being generally more abundant than SUTs and CUTs, markedly less pA^+^ and pA^−^ signal from 3′ end regions was detected for these RNA types (Figure 2D, panels 5–8). However, pA^−^ reads from gene bodies were much less biased by TU annotation (Figure 2D, panels 3 and 4), demonstrating that transcription levels vary less dramatically than the transcript output of these RNA classes. Finally, pA^+^ reads from within gene body regions were more prominent in CUT than in SUT and mRNA TUs (Figure 2D, panels 1 and 2), consistent with internal pA^+^ reads being caused by TRAMP activity. Taken together, this inspires confidence that the relatively simple division of the datasets into “gene body pA^−^” and “gene 3′ end pA^+^” signals provides a comprehensive picture of gene expression parameters for individual transcripts.

### pA^−^ 3′ End Signals Reveal RNAPII Stalling Events

**Figure 3:**
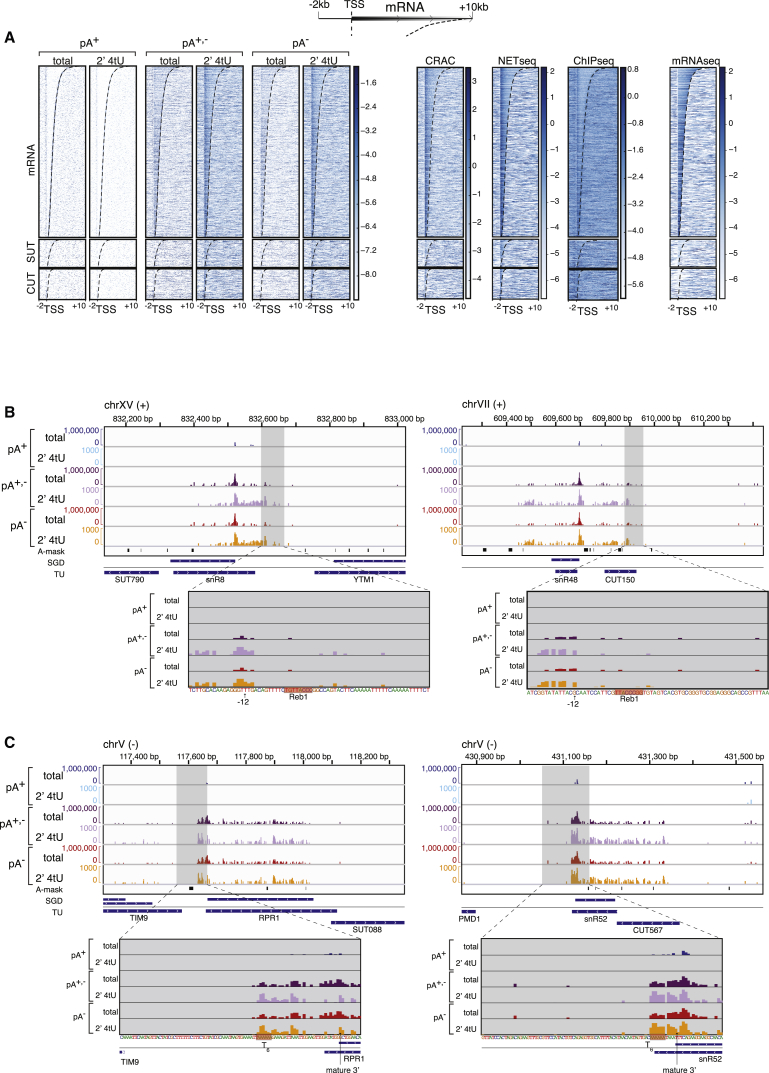
pA^−^ RNA 3′ Ends Indicate Low RNAPII Processivity at Gene 5′ Ends (A) Heatmaps as in Figure 1E but anchored to mRNA (n = 5,170), SUT (n = 847), and CUT (n = 925) TSSs and showing regions 2 kb upstream and 10 kb downstream as schematized on top. TUs were ordered by descending lengths. Read densities from RNAPII-CRAC (Milligan et al., 2016), NET-seq (Churchman and Weissman, 2011), RNAPII ChIP-seq (Warfield et al., 2017), and mRNA-seq (Churchman and Weissman, 2011) datasets are shown on the right for comparison. TES positions are marked by a dashed line. (B) Genome browser views as in Figure 1D but for genomic regions surrounding the *snR8*, *snR48* TUs. Zoom-ins of regions downstream of gene TESs are shown gray-shaded below each panel together with the DNA sequence. Reb1-binding sites are marked in red, and positions 12 bp upstream of these sites (position of the RNAPII catalytic center upstream the Reb1 roadblock) are marked with arrows. The “A-mask” track shows genomic A-rich positions selected and skipped from analysis as described in STAR Methods. (C) Genome browser views as in (B) but for genomic regions surrounding the RNAPIII-transcribed genes *RPR1* and *snR52*. T-rich stretches are marked in red, and positions of mature RNA 3′ ends are marked with arrows. See also Figure S3.

Having established that our approach offers a strong alternative to reported transcription methods, we analyzed the distribution of pA^+^ and pA^−^ reads around all interrogated TUs in more detail. Data from Figure 1E indicated that 2′ 4tU pA^−^ gene body reads were more abundant close to gene 5′ ends, which we examined further by preparing heatmaps anchored to the TSSs of mRNA, SUT, and CUT TUs. This confirmed a specific enrichment of TSS-proximal pA^−^ 3′ends for mRNA, SUT, and CUT TUs (Figure 3A, left panels) and was consistent with data collected by alternative techniques (Figure 3A, right panels). Importantly, the discernable 5′ bias of pA^−^ 3′ end signal was not caused by undue signal from surrounding short TUs (Figure S3A). Given an absence of the mammalian RNAPII stalling factor negative elongation factor (NELF) in *S. cerevisiae*, little is known about the molecular underpinnings of such 5′-biased RNAPII occupancy. We reasoned that it could also be due to premature TSS-proximal RNAPII termination, consistent with the notion that NNS-dependent and TSS-proximal terminators have been reported for a number of individual genes (Arigo et al., 2006, Creamer et al., 2011, Schulz et al., 2013, Thiebaut et al., 2008, Tuck and Tollervey, 2013). At the level of selected single genes, this was also visible in our datasets, as revealed not only by higher levels of pA^−^ 3′ ends but also by the presence of pA^+^ 3′ends, consistent with transcription termination occurring at those positions (Figure S3B). However, we found no evidence for a general accumulation of TSS-proximal pA^+^ reads (Figure 3A, left panels) and could identify numerous genes with significant 5′ bias of pA^−^ ends without detectable pA^+^ ends in the TSS-proximal region (Figure S3C). Consistently, analysis of published pA^+^ and pA^+,−^ 3′ end datasets from control and nuclear-exosome-depleted cells (Roy et al., 2016) did not suggest a general TSS-proximal and exosome-dependent pA^+^ or pA^+,−^ RNA signal (Figure S3D). It is also consistent with a previous analysis demonstrating that NNS-induced transcription termination within protein-coding genes is rare (Schulz et al., 2013).

As an alternative explanation, we considered transient RNAPII stalling at nucleosomes boundaries (Churchman and Weissman, 2011, Milligan et al., 2016). To interrogate this possibility, data were aligned to nucleosome 5′ boundaries (Jiang and Pugh, 2009), which revealed that 2′ 4tU pA^−^ 3′ ends accumulate downstream of the +1 nucleosome, with signal gradually declining further downstream (Figure S3E). We observed no discernable bias for signal accumulating upstream of nucleosome borders. Similar results were obtained when plotting published RNAPII CRAC, NET-seq, and RNAPII ChIP-seq data in the same manner (Figure S3E, bottom panels). The slightly more TSS-distal signal accumulation observed in the pA^−^ RNA-seq data compared to published datasets is likely due to removal of small RNAs owing to the library preparation technique employed (see STAR Methods). More generally, the 5′ bias of transcription in *S. cerevisiae* is likely due to lower RNAPII processivity at gene 5′ ends, which appears to be largely independent of nucleosome positioning, while premature transcription termination at these sites is limited to a restricted number of genes, as suggested previously (Schulz et al., 2013).

### pA^−^ 3′ End Signals Detect RNAP Termination Sites with Single-Nucleotide Precision

To further inquire about the usefulness of our pA^−^ 3′end data, we inspected the genome for transcripts with well-established non-polyadenylated 3′ ends. Gratifyingly, reads matching exactly the 3′ ends of mature isoforms of snRNAs and snoRNAs were orders of magnitudes more abundant in pA^+,−^ libraries than in samples, which were not treated with E-PAP (Figures S3F and S3G). The low fraction of observable pA^+^ 3′ ends (<1%) indicates an efficient quality control or complete decay of such species (Roy et al., 2016). For many snRNA and snoRNA TUs, we further observed a significant amount of 2′ 4tU pA^−^ 3′ends both up- and downstream of the 3′ end of the mature isoform (Figures 3B and S3F). These signals likely represent 3′ ends of nascent transcripts, testifying to the applicability of the approach also for these TUs. Transcription of snRNA and snoRNA TUs can be terminated at well-defined positions by so-called roadblock terminators, illustrated by the presence of the tightly bound DNA-binding protein Reb1p (Colin et al., 2014, Roy et al., 2016). Interestingly, examining the Reb1p-dependent terminators downstream of the *SNR8* and *SNR48* TUs showed a precedence for pA^−^ 3′end reads proceeding from these gene units and until the Reb1p binding site with accumulating signal ∼12 bp upstream thereof (Figure 3B). Roadblock termination at mRNA genes was also observed, as evident for the Reb1p roadblock downstream of the *RPL9B* gene (Roy et al., 2016; Figure S3H, bottom). This is consistent with RNAPII stalling and subsequent template release at this position.

A related phenotype was observed at RNAPIII TUs. RNAPIII generally terminates at homopolymeric T-stretches (Arimbasseri et al., 2013), and consistently, we observed a sharp decline of pA^−^ 3′ ends at T-runs downstream of the RNAPIII-transcribed genes *RPR1*, *SNR52*, *SCR1*, and *SNR6* (U6 snRNA) (Figures 3C and S3I). This was especially evident at *RPR1* and *SNR52* TUs, where the mature RNA 3′ ends are located upstream the T-rich regions mediating RNAPIII termination, yet the pA^−^ 3′ end signal extended to the T-runs and disappeared (Figure 3C). Thus, the presented approach can also be used to analyze features of RNAPII and III transcription at high resolution within the same experiment.

### Estimating RNA Decay Rates

**Figure 4:**
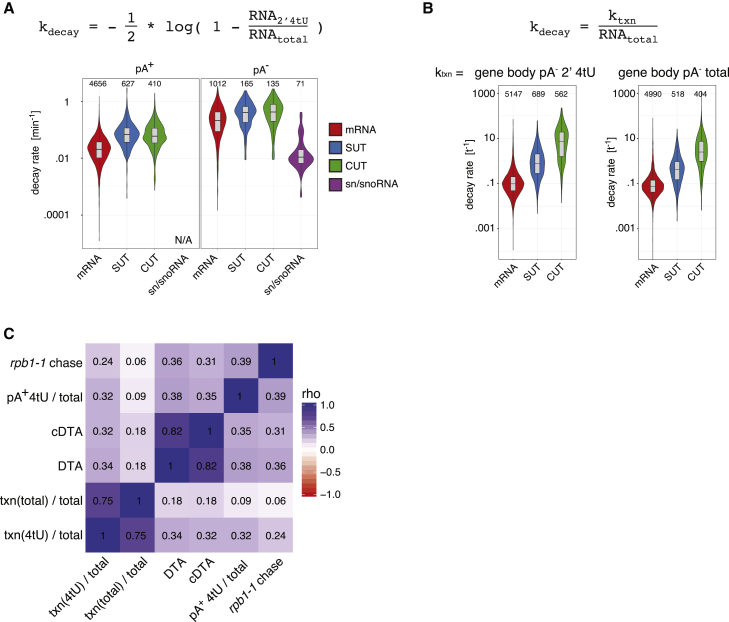
Calculated DRs Correlate with Other DR Measures (A) Violin plots depicting the distribution of DRs estimated by the ratio of 4tU to total RNA values from gene end regions using the formula shown on top (see also Figure S4A). Values for mRNA, SUT, and CUT TUs are shown separately for pA^+^-derived (left panel) and pA^−^-derived (right panel) estimates. DRs for snRNAs and snoRNAs are computed from values stemming from the exact mature 3′ ends instead of the gene end region. Estimates from pA^+^ data are not shown, as only n = 3 snRNAs and snoRNAs harbored pA^+^ signal above background in the 4tU sample at the exact mature 3′ end position. Number of TUs considered are depicted above each violin. (B) Same as in (A) but using the ratio between transcription and total RNA levels for calculation of DRs as shown on top. DRs were calculated using transcription estimates from 4tU (left panel) and total (right panel) RNA samples. Note that since transcription is measured indirectly, these data are plotted on an arbitrary scale. Number of TUs considered are depicted above each violin. (C) Spearman rank correlation (rho) matrix of DR estimates for mRNAs for which finite DR estimates exist in all depicted datasets (n = 2,528) from (A) and (B) and compared to published DR estimates from rpb1-1 chase experiments using total RNA (Presnyak et al., 2015), 4tU-based methods (dynamic transcriptome analysis [DTA]; Miller et al., 2011), and comparative DTA (cDTA; Sun et al., 2012). See also Figure S4.

In addition to interrogating transcription, the datasets presented here can also be used to estimate RNA decay rates (DRs). There are at least two independent ways to achieve this. First, a well-established method employs the relative amounts of 4tU-labeled compared to total RNA levels (Dӧlken et al., 2008; outlined in Figure S4A, top). Indeed, as we measured both pA^+^ and pA^−^ 3′ ends from 4tU and total RNA samples, DRs could be calculated independently for polyadenylated and non-polyadenylated RNA species. As expected, DRs of polyadenylated mRNAs were overall lower than those of pA^+^ SUTs and pA^+^ CUTs (Figure 4A). Moreover, pA^−^ reads from gene ends gave radically different results with much higher turnover rates, consistent with the fact that pA^−^ RNA 3′ ends are generally more abundant in the 4tU-labeled samples than in total RNA samples (Figure 2D). This is with the notable exception of mature snRNAs and snoRNAs, which have highly stable pA^−^ ends (Figure 4A). The higher turnover rates of mRNA, SUT, and CUT pA^−^ ends are likely due to the fact that these reads comprise transcription intermediates, which are by nature transient, and that post-transcriptional mRNA pA^−^ 3′ ends are commonly transient processing intermediates or highly unstable species.

An alternative approach to derive RNA DRs utilizes the fact that the amount of total RNA in a steady-state system is determined solely by transcription rates and RNA DRs. Since our data include estimates of both total RNA and transcription rates, DRs can be directly estimated (Figure 4B). Indeed, since transcription rates can be inferred from both 4tU-labeled RNA and total RNA, there are two separate ways to perform this calculation (Figure 4B, left and right panels). Gratifyingly, both yielded an overall similar result and again revealed the expected lower DRs of mRNAs as compared to SUTs and CUTs.

We tested how these different DR calculations compared to each other and to published DR estimates. As expected, most estimates, including the ones performed here, correlated positively and with high significance (Figures 4C and S4B). However, we note that DRs, derived from transcription estimates from total RNA samples, were less correlated with published DR datasets. This is most likely because nascent RNA ends are less abundant and therefore less reliably quantified in total versus 4tU RNA.

## Discussion

The results presented in this paper demonstrate that key parameters in gene expression, transcription, RNA levels, and RNA DRs can be interrogated from the same RNA sample using a set of directly comparable 3′ end RNA-seq libraries. The presented method differs technically from major existing high-throughput transcription approaches like NET-seq, RNAPII ChIP-seq, and RNAPII CRAC in that it does not require purification of RNAPII or any RNA-protein or DNA-protein cross-linking. This increases its flexibility and applicability. The feature that distinguishes the presented approach from existing 4tU-based methods is its specific detection of RNA 3′ ends to discriminate truly nascent from newly made post-transcriptional RNA. This is critical, since even short 4tU labeling times allow the production of mature RNA indistinguishable from RNAPII-connected nascent transcripts. Consequently, any 4tU-based method using RNA-seq or microarray technology, as its downstream application, cannot directly infer transcription rates. Moreover, early post-transcriptional decay may occur before or during polyadenylation, and it is not possible to dissect such events using existing protocols. For example, we previously noted a marked decrease in net mRNA production upon depletion of the nuclear pA-binding protein Nab2p (Schmid et al., 2015). However, using a conventional 4tU-based protocol, it was not possible to distinguish a transcriptional from a post-transcriptional cause of the phenotype (Schmid et al., 2015), whereas the 3′ end sequencing approach presented here seamlessly permits this (Tudek et al., 2018; in this issue of *Cell Reports*). Moreover, this approach further allows for a precise analysis of RNAPII density along the genome, mapping for example its positions of stalling at gene 5′ ends and at roadblock terminators.

We consider 2′ 4tU pA^−^ 3′ends inside of gene bodies as representative of nascent transcript 3′ ends. A potential complication with this assumption is that such 3′ ends could in principle also be produced by RNA decay. Such activity occurring after RNA purification is typically conducted by contaminating RNase A enzyme, producing 3′ phosphate (3′P) ends, or by hydrolytic cleavage, leaving 3′P/2′OH or 3′OH/2′P ends (Cuchillo et al., 2011). 3′P-marked ends do not serve as substrates for *E. coli* PAP and would therefore go undetected, while it is unclear whether 3′OH/2′P ends are recognized (Balbo and Bohm, 2007). In contrast, pA^−^ 3′ends produced *in vivo* by post-transcriptional processing or 3′–5′ decay would readily be detected by our analysis. However, for the RNA samples analyzed here, this does not appear to be a major source of pA^−^ 3′ends. This is because we detect an increased presence of such 3′ ends in the 2′ 4tU fraction, their equal coverage between exons and introns of pre-mRNAs, and their discernable presence downstream of gene TESs. Moreover, pA^−^ 3′end densities are very similar to NET-seq and RNAPII CRAC signal patterns. This is evidenced by the correlation of gene body signals and the similar metagene profiles between the different methodologies. We therefore surmise that 2′ 4tU RNA 3′OH ends largely reflect RNAP transcription.

Our data reveal that pA^−^ 3′ ends are also readily detectable in total RNA preparations, although at a lower level than in the 4tU-labeled samples. Somewhat unexpectedly, these 3′ ends behave, in many respects, quite like labeled pA^−^ 3′ ends. This is apparent from inspections of highly expressed mRNA genes and from their good correlation with RNAPII-NET-seq, RNAPII-CRAC, and RNAPII-ChIP data, suggesting that total pA^−^ gene body reads are largely indicative of transcription. This result is in line with a large body of research indicating that decay of cytoplasmic mRNA occurs via 5′–3′ degradation with little contribution by 3′–5′ decay (Parker, 2012). Even so, differences between total and 2′ 4tU RNA were also noted. For intron-containing RNAs, the intron-to-exon signal ratio was roughly 2-fold higher in the 2′ 4tU-labeled sample than in the total sample. Moreover, EE junction reads were more prevalent in total compared to 2′ 4tU RNA. While it is formally possible that this reflects a special behavior of intron-containing RNAs (they could be more susceptible to 3′–5′ decay), a more likely explanation is that 3′–5′ decay intermediates contribute a more noticeable amount of reads in total RNA samples. Still, the overall correlation between pA^−^ 3′ ends of total and labeled samples, and the correlation of both of these with published transcription data, demonstrate that even total RNA samples can be useful for estimating transcription levels. The lower pA^−^ coverage in total RNA is less worrisome, since samples presented here were sequenced at relatively low depths. Taken together, the presented method should be readily adaptable to a wide range of experimental setups and perhaps especially relevant when RNA is available but measurement of transcription is challenging.

## STAR★Methods

### Key Resources Table

**Table:** 

REAGENT or RESOURCE	SOURCE	IDENTIFIER
**Chemicals, Peptides, and Recombinant Proteins**		

MTSEA-biotin	Biotium	Cat#90064
Rapamycin	Cayman chemicals	Cat#13346
4tU	Aldrich	Cat#440736-1G

**Critical Commercial Assays**		

Turbo DNase free kit	Ambion	Cat#AM1907M
*Escherichia coli* poly(A) polymerase kit	ThermoFisher	Cat#AM1350
PureLink micro RNA purification kit	Ambion	Cat#12183018A
Ribo-Zero Gold rRNA Removal kit for yeast	Ilumina	Cat#MRZY1306
RiboLock Rnase Inhibitor	ThermoSceintific	Cat#E00381
Lexogen QuantSeq 3′ mRNA-Seq Library Prep Kit REV	Lexogen	Cat#016.96
Dynabeads MyOne Streptavidin C1	Invitrogen	Cat#65002
Zeba Spin Desalting Columns 7KMWCO	Thermo Scientific	Cat#89890

**Deposited Data**		

2 min 4tU	This paper	GEO: GSE108550

**Experimental Models: Organisms/Strains**		

*S. cerevisiae* genome	N/A	UCSC: sacCer3
*S. pombe* genome	N/A	ENSEMBL: EF2
Mex67-AA (‘strain 1’)	Haruki et al., 2008; Euroscarf	*as W303, tor1-1 fpr1::NAT RPL13-2xFKBP12::TRP1 MEX67-FRB::kanMX6*
Nab2-AA (‘strain 2′)	Schmid et al., 2015	*as W303, tor1-1 fpr1::loxP-LEU-loxP RPL13-2xFKBP12::TRP1 Nab2p-FRB::HIS3*

**Software and Algorithms**		

BBMAP v 35.92	unpublished	https://jgi.doe.gov/data-and-tools/bbtools/
STAR aligner v GitHub 2016-03-14	Dobin et al., 2013	https://github.com/alexdobin/STAR
samtools v 1.3	Li et al., 2009	http://www.htslib.org/
HTSeq v 0.6.0	Anders et al., 2015	https://pypi.python.org/pypi/HTSeq
R package DESeq2 v 1.10.1	Love et al., 2014	http://bioconductor.org/packages/release/bioc/html/DESeq2.html
deepTools2 software suite v2.2.4		http://deeptools.readthedocs.io/en/latest/index.html

**Other**		

Code used for this publication	This paper	https://github.com/manschmi/MexNab_3seq
RNAPII ChIP-tiling array	Mayer et al., 2010	ArrayExpress: E-TABM-1033
RNAPII ChIP-seq	Warfield et al., 2017	sample GSM2551210 from GEO: GSE97081
NET-seq	Churchman and Weissman 2011	sample GSM617027 from GEO: GSE25107
RNA-seq	Churchman and Weissman 2011	sample GSM617028 from GEO: GSE25107
RNAPII CRAC	Milligan et al., 2016	sample GSM1706520 from GEO: GSE69676
cDTA	Sun et al., 2012	ArrayExpress: E-MTAB-760
DTA	Miller et al., 2011	ArrayExpress: E-MTAB-439
*rpb1-1* chase	Presnyak et al., 2015	GEO: GSE57385
RNA-seq	Schmid et al., 2015	SRA: SRX844447

### Contact for Reagent and Resource Sharing

Further information and requests for resources and reagents should be directed to and will be fulfilled by the Lead Contact, Torben Heick Jensen (thj@mbg.au.dk).

### Experimental Model and Subject Details

#### Yeast strains and growth conditions

All experiments were done with strains Mex67-AA (strain 1) and Nab2-AA (strain 2) described in (Haruki et al., 2008) and (Schmid et al., 2015), respectively. Genotypes and origin of those strains are described in the Key Resource Table. These strains were not authenticated. Cells were transformed with a 2μ plasmid, carrying the URA marker and driving overexpression of the uridine permease FUI1 (Barrass et al., 2015). For experiments described here, cells were cultivated in –URA drop-out medium at 30°C.

### Method Details

#### 4tU labeling and RNA purification

The 4tU labeling and purification method was from (Barrass et al., 2015) with minor modifications. Briefly, we used 50 mL of cells grown exponentially (OD 0.4 – 0.6) in minimal medium lacking uracil. Cells were labeled by addition of 100 μM (w/v final) 4tU (4-thiouracil, Sigma, from a 100 mM stock in DMSO) and, after incubation for 2 min, quenched by pouring cultures into flasks, containing 1x volume of ethanol pre-chilled on dry ice. Cells were then pelleted by spinning for 3 min at 4’000 rpm at 4°C and pellets washed with ice-cold water. RNA was purified using the conventional hot-phenol extraction method and resuspended in 300 μl of RNA storage solution (Ambion). Then RNA concentration was measured and a 1/100 w/w of 4tU labeled *S. pombe* spike-in RNA was added. The spike-in RNA represented total RNA from *S. pombe* cells grown exponentially in YPAD medium at 30°C and labeled with 5 mM of 4tU for 10 min.

For biotin labeling 300 μg of total RNA was coupled using 10 μl of 5 mg/ml MTSEA-biotin in DMF (Biotium) in a 400 μl reaction buffered with 10 mM HEPES-KOH pH 7.5 and 1 mM EDTA for 30’ at 25°C in the dark. Reactions were then poured onto Zeba desalting columns (Zeba Spin Desalting Columns, 7K MWCO, 2 ml, ThermoFisher Scientific), washed and drained 3x in RNA storage solution. Subsequently, an additional 130 μl of RNA storage solution was applied and the flow-through was collected. RNA was extracted once with phenol:chloroform and precipitated from the desalted reactions using NaOAc and ethanol and resuspended in 250 μl RNA storage solution. An 8 μl aliquot was collected at this step for total RNA analysis. Biotinylated RNA was then purified using magnetic streptavidin-coated beads (Streptavidin Magnetic Beads Dynabeads MyOne C1, Invitrogen), and 240 μl of the RNA samples were diluted to 300 μl in 0.1 M Na-PO_4_ pH 6.8; 10 mM Tris-HCl pH 7; 0.2 M NaCl; 25 mM MgCl_2_; 0.1% SDS. 30 μl of magnetic beads were equilibrated in the same buffer and blocked by incubating with 200 μg of glycogen in the same buffer for 20 min at room temperature (RT). Beads were drained, the RNA solution was added to the beads and the reaction was incubated for 30 min at RT with gentle agitation. Then supernatant was removed and beads were washed 5x with 400 μl of binding buffer, 1x with 10 mM Tris-HCl pH 7.5; 0.5 mM EDTA; 1 M NaCl, and bound RNA eluted 2x for 5 min using 50 μl 0.7 M beta-mercaptoethanol at RT under gentle agitation. Eluates were combined and RNA was precipitated overnight using NaOAc and ethanol with 100 μg of glycogen as a carrier. After final washing, RNA was resuspended in 20 μl of RNA storage solution. RNA quality and concentration were measured using a Bioanalyzer and the RNA pico kit (Agilent). 4 μl (6 μg) of the total RNA aliquot and 10 μl (2 – 10 ng) of the 4tU IP samples were then used for a regular 24 μl TURBO DNase (Ambion) reaction.

#### *In vitro* polyadenylation

5 μl of TURBO DNase treated RNA was polyadenylated with *E. coli* PAP (E-PAP, ThermoFisher Scientific) in 20 μl reactions, containing 1x reaction buffer, 2.5 mM MnCl_2_; 0.4 U E-PAP and 0.8 U RiboLock RNase inhibitor (ThermoFisher Scientific) for 30 min at 30°C. Reactions were then purified using a PureLink micro RNA purification kit (Ambion) and eluted using 22 μl RNase-free water. PureLink eluates were rRNA depleted using a down-scaled reaction of the Ribo-Zero Gold rRNA Removal Kit for Yeast (Illumina). That is, 18 μl of RNA was incubated with 2 μl of reaction buffer, 2 μl of removal solution and 8 μl of water for 10 min at 65°C, and put to RT for 5 min. This reaction was cleared using 65 μl of rRNA magnetic beads for 5 min at RT and 5 min at 50°C. Final samples were precipitated using NaOAc and ethanol with 20 μg of glycogen as carrier and resuspended in 10 μl of RNA storage solution.

#### Library preparation and sequencing

1 μl of input diluted to 5 μl using water and 5 μl of IP samples were used for preparation of QuantSeq REV libraries (Lexogen GmbH, Vienna, Austria). Libraries were prepared according to the manufacturer’s specifications and sequenced at Lexogen Vienna Biocenter Core Facilities multiplexing 32 samples on a HiSeqV4 SR50 run and using the QuantSeq REV specific primer CSP. All samples were prepared from both yeast strains, and 4tU samples subjected to *in vitro* polyadenylation were prepared from 3 independent cultures of each yeast strain. An overview of samples, read quality control and mapping statistics are provided in Table S1.

#### Quality Control, Filtering and Mapping

Barcode splitting and supply of final data as unmapped bam files was provided by Lexogen. We then applied quality filtering, read trimming and the mapping strategy recommended for QuantSeq REV data. In brief, unmapped bam files were converted to fastq using bamToFastq functions from bedtools (v2.16, (Quinlan and Hall, 2010)). Adapters were trimmed using the bbduk.sh script from BBMAP software (v35.92, https://jgi.doe.gov/data-and-tools/bbtools/) using parameters k = 13; ktrim = r; useshortkmers = t; mink = 5; qtrim = t; trimq = 10; minlength = 20; ref = /home/schmidm/annotations/common/Lexogen_adapters_with_pA.fa.gz. The Lexogen adapters file contains the common Illumina TruSeq adaptor sequences 1-27 and an additional A18 sequence to trim homopolymeric A-tails often observed in that protocol (see also Table S1). Trimmed reads were then mapped using STAR aligner (GitHub v2016-03-14, (Dobin et al., 2013)) together with samtools (v1.3, (Li et al., 2009)) at settings–outFilterType BySJout;–outFilterMultimapNmax 20;–alignSJoverhangMin 8;–alignSJDBoverhangMin 1;–outFilterMismatchNmax 999;–alignIntronMin 20;–alignIntronMax 2000;–outSAMtype BAM SortedByCoordinate. The index file used was derived from the catenation of fasta files from yeast genomes *S. cerevisiae* genome release sacCer3 and *S. pombe* genome ENSEMBL release EF2. The merged genome was indexed for STAR using settings–runMode genomeGenerate;–sjdbGTFfile r64_2_1.gff3;–sjdbGTFtagExonParentTranscript Parent;–sjdbGTFfeatureExon CDS;–genomeSAindexNbases 11;–sjdbOverhang 49. That is, providing only splice-junction information for the *S. cerevisiae* genome sacCer3. Bam files were then indexed using samtools and 5′end positions (marking the RNA 3′ ends) of uniquely aligned reads were obtained using a custom python script using HTSeq (v0.6.0, (Anders et al., 2015)) that computes coverage of uniquely mapping 5′ ends in bedgraph format (see code). Trimming and mapping statistics were obtained from the output of the bbduk script and the log files from the STAR mapper, respectively.

#### Genomic Adenosine masking

End positions likely deriving from priming of the reverse transcription primer to endogenous A-rich sequences were removed from analysis. Criteria for removal were based on (Roy et al., 2016). In brief, 2 types of genomic positions were removed: (1) those containing more than 4 As and no Cs or Ts within 6 bp downstream, and (2) those containing more than 12 As in a 18 bp window downstream. Note that Roy et al. also filtered for more than 15 As in a 18 bp window upstream, which is supposed to remove artifacts due to mismapping (and not priming to genome-encoded A stretches). However, inspection of our data did not reveal a significant presence of such reads and this 3^rd^ filter was therefore not applied. Genomic positions fitting these criteria were collected in bed file format and positions removed from track files using bedtools function subtract (Quinlan and Hall, 2010).

#### Normalization to *S. pombe* spike-ins

3′ ends mapping to *S. pombe* transcripts (TSS to TES + 300 bp) were counted using a custom script based on bedtools (see code). This was used to derive scaling factors by the sizeFactors function from the R package DESeq2 (v1.10.1, (Love et al., 2014)), using otherwise default settings. These sizeFactors were then used to scale the *S. cerevisiae* -derived signal in the bedgraph track files from above.

#### Background subtraction for 4tU samples

We reasoned that the short 4tU labeling time of 2 min might cause contamination of the purified fraction with unlabeled RNA (termed ‘background’ below). To address this worry, mock (negative) control IP samples were obtained by processing total RNA from cells harvested without prior incubation with 4tU in the growth medium. This sample was prepared in parallel with the corresponding experiment and, importantly, contained the same amount w/w total RNA of spike in RNA. Since the spike-ins were also partially 4tU labeled, data normalized to these yielded direct information about background. *S. pombe*-normalized signal from negative control IP samples was subtracted from 2′ 4tU samples to obtain background-subtracted (BGsub) signals and only those values used for analysis shown in the main text. Features or genomic intervals where BGsub values were ≤ 0 (ie background was equal to or higher than the experimental value) were omitted in all Figures. Analysis of the mock samples showed that the negative IP samples generally resembled total RNA, and not 4tU IP, samples concerning their relative coverage of gene body versus end regions, their coverage of introns and of snRNA and snoRNA TUs (data not shown). Spike-in normalized counts from 4tU IP samples were above negative control IP counts for the majority of investigated TUs. This enrichment was especially pronounced in the gene body regions in the pA^+,-^ datasets, consistent with the transient nature of those tails.

#### Generation of pA minus data

To obtain an estimate of pA^-^ signal at each position, we subtracted the *S. pombe* normalized pA^+^ signal from the pA^+^ and pA^-^ signal. For the 2′ 4tU IPs this was done after background subtraction.

#### Annotations

mRNA, SUT and CUT annotations were from (Xu et al., 2009) lifted to UCSC sacCer3. Mature 3′ end positions for sn-/snoRNAs were from the SGD annotation for sacCer3.

#### Genome browser tracks

All tracks were produced with log-scaled y-axes, except for the ChIP-tiling array data, which was shown on a linear scale.

#### Counting signals of gene body and end regions

Gene body regions were defined to derive from the TU TSS to 200 bp upstream of the TES (TSS to TES - 200 bp). Gene end regions were defined to derive from 200 bp upstream to 200 bp downstream the annotated TES (TES ± 200 bp). Signal within each region was counted using bigWigAverageOverBed from UCSC tools (v2 (Kent et al., 2010)) using *S. pombe* normalized, background-subtracted pA^+^ or pA^-^ bigwig track files. Counts were then analyzed using custom scripts in R. Average counts across two strains and replicates (see Table S1 for availability) were used for further analysis unless otherwise stated.

#### Decay rates

DRs were derived from 4tU to total RNA ratios using the formula DR = (1/time)^∗^-log(1 – RNA_4tU_/RNA_total_), where time = 2 min, ‘RNA_4tU_’ represents the normalized and background subtracted reads of gene ends (either pA^+^ or pA^-^) from the 2′ 4tU samples and ‘RNA_total_’ represents the corresponding normalized reads from total RNA. For calculating DRs by comparing transcription to total RNA levels, we used gene body read densities (i.e., normalized and background subtracted 4tU reads or normalized total RNA reads) scaled by effective gene body length (i.e., gene body length subtracted for A-filtered positions) and total RNA were normalized gene end reads from total RNA as above.

#### Metagene profiles

Heatmaps and metagene profiles were obtained from the representative replicate 1 of strain 1 using computeMatrix and plotHeatmap functions from the deepTools2 software suite v2.2.4 (Ramírez et al., 2014).

#### Splice junction read analysis

Reads overlapping EE, EI and IE junctions were counted in all raw bam files using a custom python script and the module HTSeq (v0.7.2). EE reads were those reads overlapping an annotated exon-exon junction and mapping to at least 2nt on each side without mapping to intronic positions. EI and IE were those reads mapping across an exon-intron junction with at least 2 nt mapping on each side. Junction read counts were then normalized using *S.pombe* based size factors (see above) and for 2′ 4tU IP samples background from the mock control IP was subtracted. Quantification of EE, EI and IE reads was applied to a control RNA-seq dataset (SRA: SRX844447) derived from a similar strain background in unperturbed cells. Those data are presented as raw reads without further processing.

#### Transcription estimates from published datasets

For NET-seq, RNA-seq, ChIP-seq and ChIP-tiling experiments available coverage data were used (see Key Resources Table). For RNAPII CRAC, mapped reads were converted to coverage tracks using custom scripts. All data were lifted to genome release sacCer3 if necessary and otherwise handled and plotted using the strategy applied also for our 3′end seq data as outlined above.

#### Published decay rate estimates

Transcription- and decay-rates were obtained from (Miller et al., 2011 (DTA)), (Sun et al., 2012 (cDTA)) and (Presnyak et al., 2015 (*rpb1-1* chase)). Half-life and decay estimates were obtained from the Supplemental Information of the relevant publications. In cases where only half-life information was provided, decay-rates were obtained using the equation k = ln(2)/halflife.

### Quantification and Statistical Analysis

Correlation matrices depict spearman rank correlations of log_2_-transformed average values. Those were obtained from R function ‘cor’ using a matrix of log_2_ values, method = ’spearman’ and use = ’pairwise complete observations’ as input. P values were obtained from R function cor.test and plotted as ‘–log10(p)’ for the spearman rank correlation values. Dendrograms were computed from spearman rank correlation matrices, transformed to distance measure using ‘ dist = (1 – rho^spearman^)/2 ’ and then clustered using the R built-in function ‘hclust’ with default settings.

### Data and Software Availability

The accession number for the data from 2′ 4tU labeling experiments reported in this paper is GEO: GSE108550. Published datasets were obtained from the following sources: NET-seq: GEO: GSM617027; RNA-seq: GEO: GSM617028; RNAPII CRAC: GEO: GSM1706520; RNAPII ChIP-seq GEO: GSM2551210 and RNAPII ChIP-tiling: ArrayExpress:E-TABM-1033 (processed data for sample: Rpb3_InputAndMockNormalized). Junction reads analysis of regular RNA-seq are based on SRA: SRX844447. All relevant code and analysis scripts are available at GitHub (https://github.com/manschmi/MexNab_3seq).

## Supplemental Information

Document S1. Figures S1–S4 and Table S1

Document S2. Article plus Supplemental Information
