# Supplementary material for: Simultaneous Measurement of Transcriptional and Post-transcriptional Parameters by 3′ End RNA-Seq
Source: Cell Rep. 2018 Aug 28;24(9):2468–2478.e4. doi: 10.1016/j.celrep.2018.07.104 (PMC6130049; doi:10.1016/j.celrep.2018.07.104)
Supplement: Document S1. Figures S1–S4 and Table S1 [file mmc1.pdf]

Cell Reports, Volume *24*

Supplemental Information

Simultaneous Measurement of Transcriptional and Post-transcriptional Parameters by 3′ End RNA-Seq

Manfred Schmid, Agnieszka Tudek, and Torben Heick Jensen

Figure S1

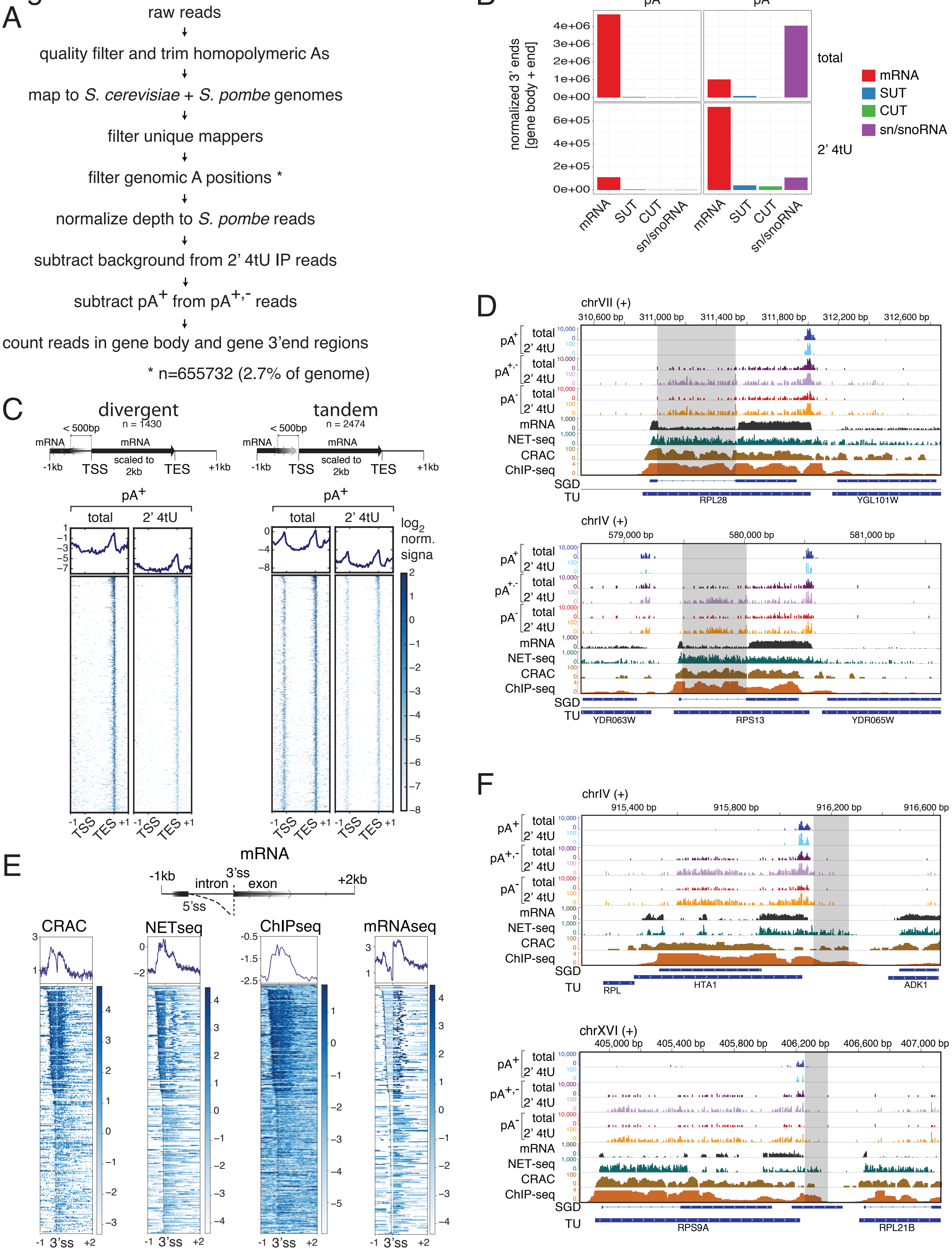
A
raw reads
quality filter and trim homopolymeric As
map to S. cerevisiae + S. pombe genomes
filter unique mappers
filter genomic A positions *
normalize depth to S. pombe reads
subtract background from 2' 4tU IP reads
subtract pA+ from pA+,- reads
count reads in gene body and gene 3'end regions
* n=655732 (2.7% of genome)
B
pA+
pA−
total
2' 4tU
normalized 3' ends [gene body + end]
mRNA
SUT
CUT
sn/snoRNA
C
divergent
n = 1430
tandem
n = 2474
< 500bp
mRNA
scaled to 2kb
-1kb
TSS
TES
+1kb
pA+
total
2' 4tU
log2 norm. signa
D
chrVII (+)
310,600 bp
311,000 bp
311,400 bp
311,800 bp
312,200 bp
312,600 bp
pA+
pA+,-
pA-
mRNA
NET-seq
CRAC
ChIP-seq
SGD
TU
RPL28
YGL101W
chrIV (+)
579,000 bp
580,000 bp
581,000 bp
YDR063W
RPS13
YDR065W
E
mRNA
-1kb
intron
3'ss
exon
+2kb
5'ss
CRAC
NETseq
ChIPseq
mRNAseq
F
chrIV (+)
915,400 bp
915,800 bp
916,200 bp
916,600 bp
RPL
HTA1
ADK1
chrXVI (+)
405,000 bp
405,400 bp
405,800 bp
406,200 bp
406,600 bp
407,000 bp
RPS9A
RPL21B

Figure S1, related to Figure 1

(A) Workflow of the utilized bioinformatics pipeline. Raw RNA-seq reads were first filtered to remove low-quality reads and trim away A-stretches, a typical artifact when employing the library preparation kit. Remaining reads were then mapped to merged *S. cerevisiae* and *S. pombe* genomes. Only unique mappers were considered and reads mapping to genomic positions upstream A-rich regions, likely deriving from internal RT priming, were ignored. Reads mapping to *S. pombe* were counted and used for normalization purposes. Finally, negative control IP (background) values were subtracted from the 2' 4tU IP samples and pA^{+} signal was subtracted from $pA^{+,-}$ signal to generate pA^{-} data. Signal from gene body- and end-regions was counted to estimate transcription, RNA production and RNA levels as explained in the main text. See Materials and Methods for details.

(B) Amount of 3'ends mapping to the different indicated TU annotation classes. Shown are sums of *S. pombe* normalized signals for TU TSSs to TESs+200bp. For 2' 4tU IPs, background is subtracted.

(C) Metagene profiles and heat maps as in Figure 1E, but distinguishing mRNAs with an upstream gene less than 500bp from their TSSs either on the opposite strand ('divergent', left panels, n=1429) or on the same strand ('tandem', right panels, n=2473) as schematized on top. Only pA^{+} data are shown.

(D) Genome browser views as in Figure 1D, but for intron-containing mRNAs *RPL28* and *RPS13*. Tracks for mRNA (Churchman and Weissman, 2011), NET-seq (Churchman and Weissman, 2011), RNAPII CRAC (Milligan et al., 2016), and RNAPII ChIP-seq (Warfield et al., 2017) experiments are included for comparison. Intronic regions are shaded gray.

(E) Heat maps as in Figure 1F, but showing read densities from RNAPII CRAC (Milligan et al., 2016), NET-seq (Churchman and Weissman, 2011), RNAPII ChIP-seq (Warfield et al., 2017) and mRNA-seq (Churchman and Weissman, 2011) datasets.

(F) Genome browser views as in Figure S1D, but for mRNAs *HTA1* and *RPS9A*. Regions with discernable pA^{-} signal downstream major pA^{+} signals are shaded gray.

Figure S2

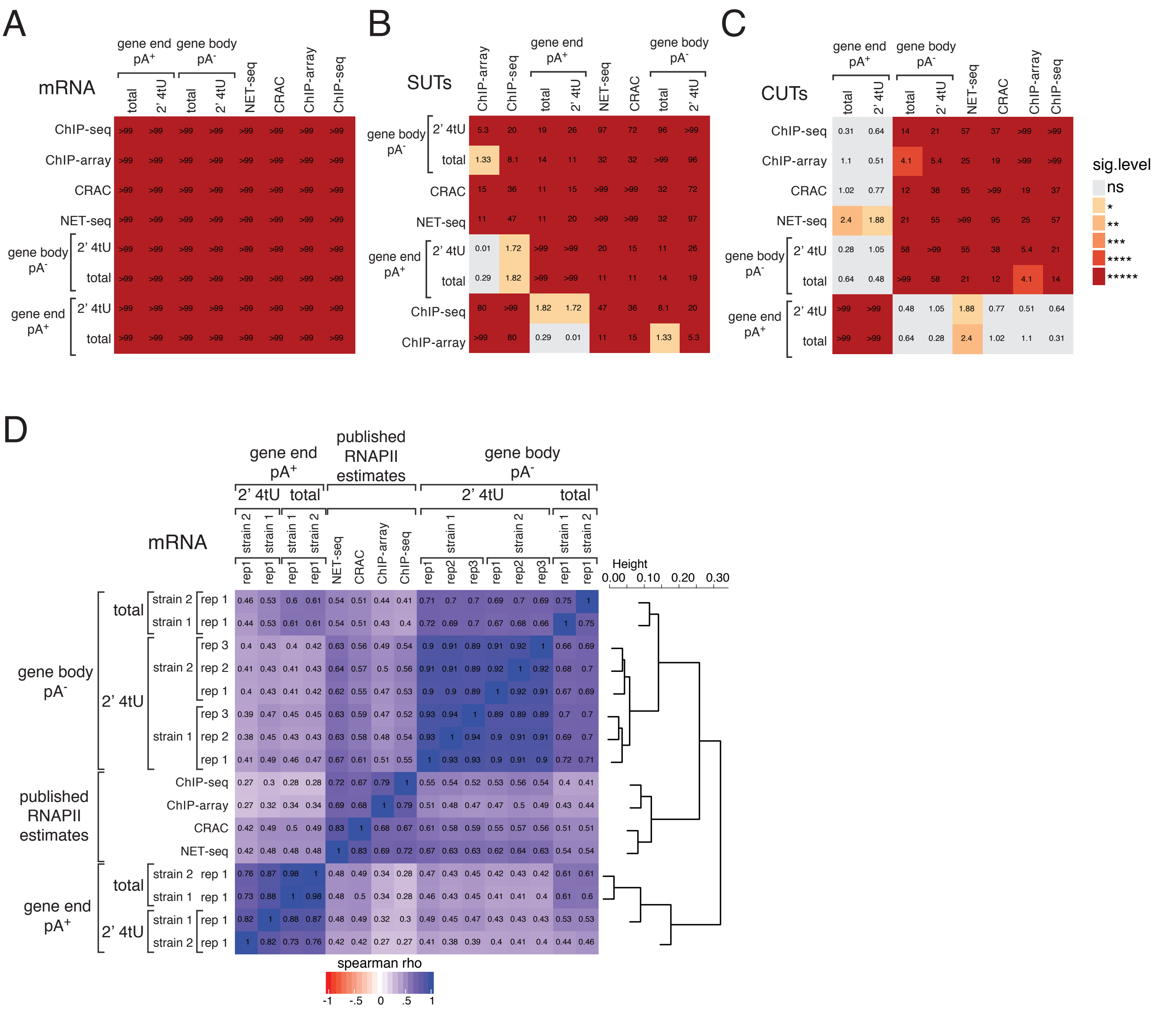

A
mRNA
gene end pA+
gene body pA-
total
2' 4tU
NET-seq
CRAC
ChIP-array
ChIP-seq
B
SUTs
C
CUTs
sig.level
ns
*
**
***
****
*****
D
published RNAPII estimates
strain 1
strain 2
rep1
rep2
rep3
rep 1
rep 2
rep 3
Height
0.00
0.10
0.20
0.30
spearman rho
-1
-.5
0
.5
1

Figure S2, related to Figure 2

(A-C) Significance of correlations shown in Figure 2A-C using $-\log_{10}$(p-value) of the spearman rank correlations. Color-codes are 'ns' (not significant, $p >= .05$), '*' ($p < .05$, $-\log_{10}(p) > 1.3$), '**' ($p < .01$, $-\log_{10}(p) > 2$), '***' ($p < .001$, $-\log_{10}(p) > 3$), '****' ($p < .0001$, $-\log_{10}(p) > 4$), '*****' ($p < .00001$, $-\log_{10}(p) > 5$).

(D) Spearman rank correlation (rho) matrix and hierarchical clustering for mRNAs as in Figure 2A except that values from the 2 independent strains, and for the pA^- 2' 4tU samples for each of the 3 biological replicates, are used separately to demonstrate experimental reproducibility.

Figure S3

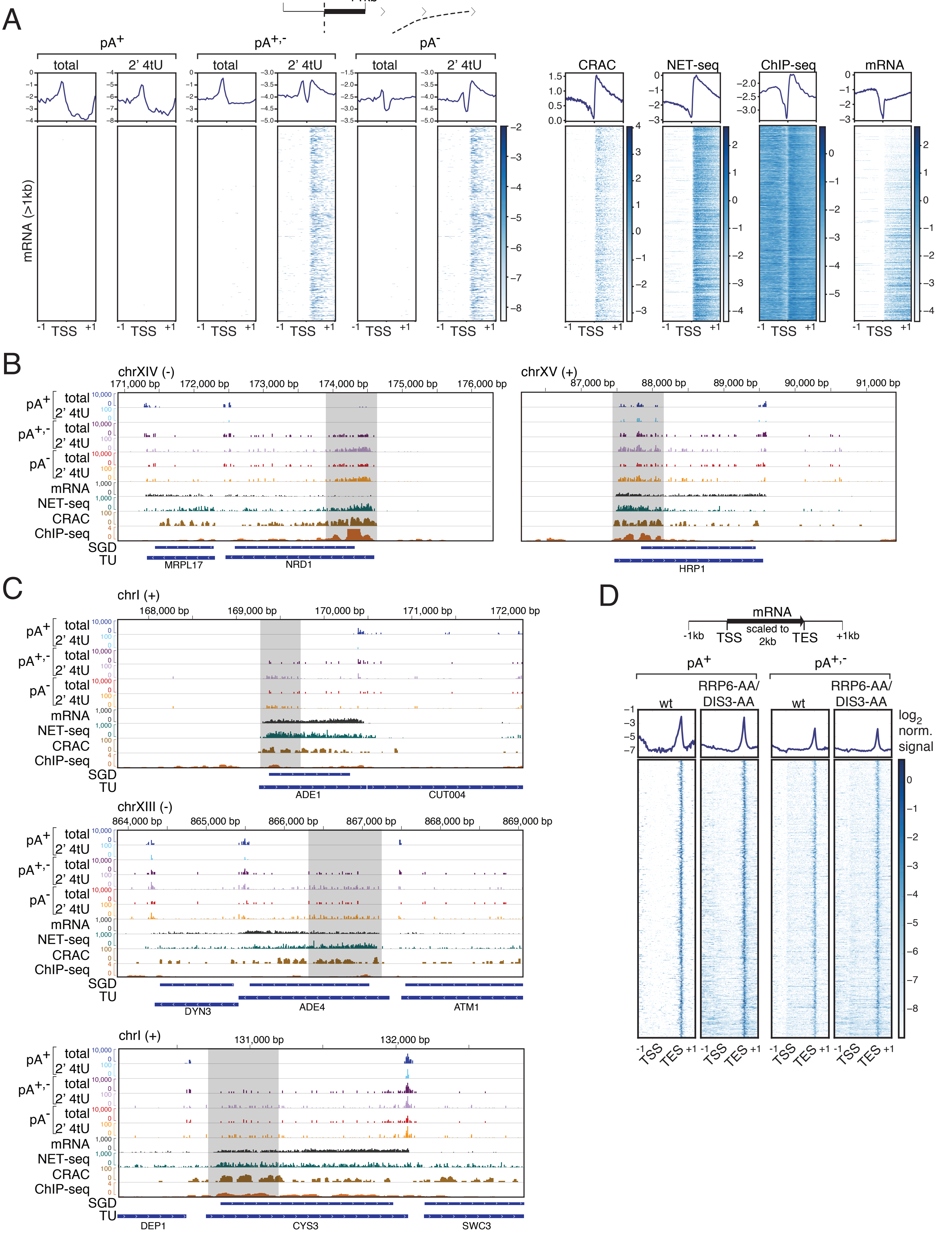
A
mRNA > 1kb
-1kb
TSS
+1kb
pA+
pA+,-
pA-
total
2' 4tU
CRAC
NET-seq
ChIP-seq
mRNA
mRNA (>1kb)
-1 TSS +1
B
chrXIV (-)
171,000 bp
172,000 bp
173,000 bp
174,000 bp
175,000 bp
176,000 bp
chrXV (+)
87,000 bp
88,000 bp
89,000 bp
90,000 bp
91,000 bp
SGD
TU
MRPL17
NRD1
HRP1
C
chrI (+)
168,000 bp
169,000 bp
170,000 bp
171,000 bp
172,000 bp
ADE1
CUT004
chrXIII (-)
864,000 bp
865,000 bp
866,000 bp
867,000 bp
868,000 bp
869,000 bp
DYN3
ADE4
ATM1
131,000 bp
132,000 bp
DEP1
CYS3
SWC3
D
mRNA
scaled to 2kb
-1kb
TSS
TES
+1kb
wt
RRP6-AA/ DIS3-AA
log2 norm. signal
-1 TSS TES +1

Figure S3

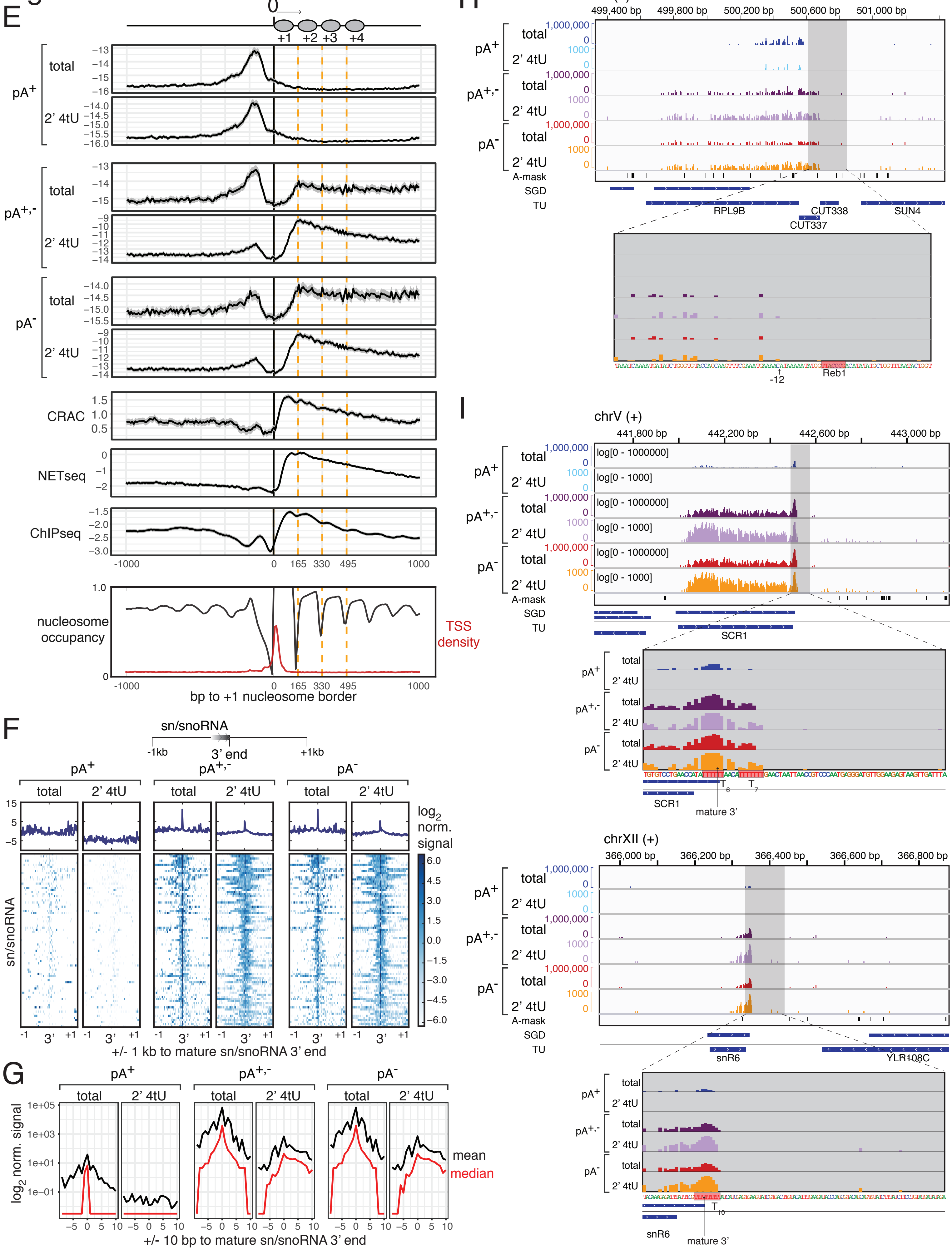
E
F
G
H
I
pA+
pA+,-
pA-
total
2' 4tU
CRAC
NETseq
ChIPseq
nucleosome occupancy
TSS density
bp to +1 nucleosome border
sn/snoRNA
3' end
-1kb
+1kb
log2 norm. signal
+/- 1 kb to mature sn/snoRNA 3' end
mean
median
+/- 10 bp to mature sn/snoRNA 3' end
chrXIV (+)
A-mask
SGD
TU
RPL9B
CUT338
CUT337
SUN4
Reb1
-12
chrV (+)
SCR1
mature 3'
chrXII (+)
snR6
YLR108C

Figure S3, related to Figure 3

(A) Metagene profiles and heatmaps as in Figure 1E and S1E, but shown for the region +/- 1kb around the TSS for mRNA genes longer than 1kb (n=3970). Genes were sorted by length.

(B) Genome browser views as in Figure S1D, but for genomic regions surrounding the *HRP1* and *NRD1* TUs. Regions of ncRNA TUs overlapping gene 5' ends are shaded gray.

(C) Genome browser views as in Figure S1D, but for genomic regions surrounding genes showing marked 5' bias in pA^{-} signals.

(D) Metagene profiles and heat maps for pA^{+} and $pA^{+,-}$ read data from total RNA samples of control and Rrp6/Dis3 doubly-depleted cells from (Roy et al., 2016). Shown are data for divergent mRNA TUs (n=1429) as in Figure S1B, left panels, to avoid undue biases by read-through signals from upstream tandem TUs.

(E) Metagene profiles showing means of $\log_2$ read densities at each position for regions 1kb up- and downstream of the upstream boundary of +1 nucleosomes (schematized on top) as defined by (Jiang and Pugh, 2009) and for mRNA genes longer than 1kb (n=2025). Displayed samples are anchored around the 5'border of the +1 nucleosome relative transcription direction. Vertical lines indicate similar borders of +2, +3 and +4 nucleosomes. The sharp decline of pA^{-} signal upstream the +2 nucleosome is most likely caused by the employed counterselection of small RNA species, whereas the pronounced accumulation of pA^{+} ends upstream of the +1 nucleosome, stem from PAS of tandem upstream genes (data not shown, see also Figure S1B). Bottom panels show density of nucleosomes and mRNA TSSs relative to the same positions for comparison.

(F) Metagene profiles and heat maps as in Figure 1E, but for regions 1kb up- and down-stream of mature sn/snoRNA 3'ends (n=83) as schematized on top.

(G) Metagene profiles as in (A), but showing mean and median values at each single nt positions for regions 10bp up- and down-stream of mature sn/snoRNA (n=83) 3' ends.

(H) Genome browser views as in Figure 3C but for *RPL9B*.

(I) Genome browser views as in Figure 3C, but for genomic regions surrounding the RNAPIII-transcribed genes *SCR1* and *snR6*.

Figure S4

A

$$k_{decay} = - \frac{1}{t} * \log(1 - \frac{RNA_{4tU}}{RNA_{total}})$$

$$k_{decay} = \frac{k_{txn}}{RNA_{total}}$$

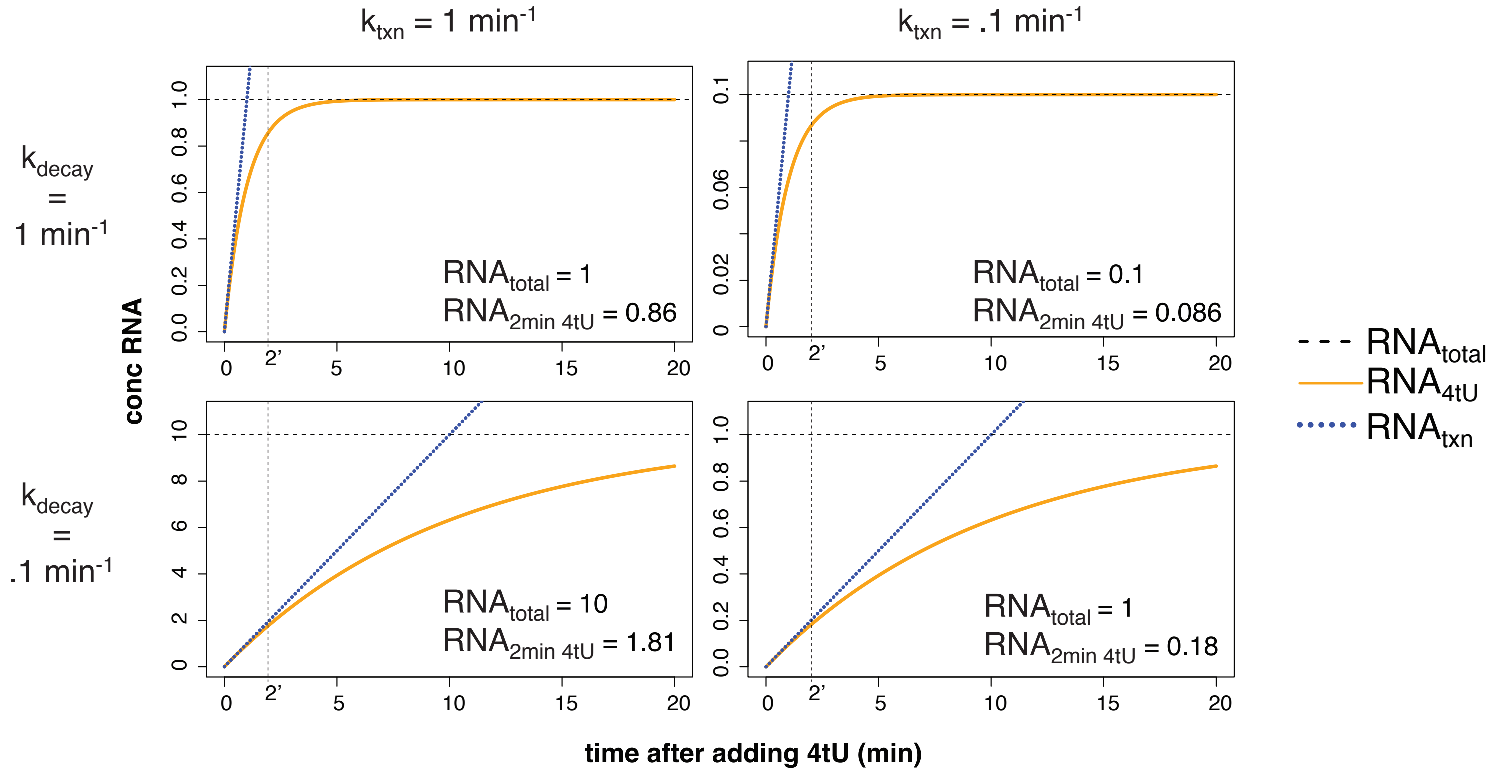

B

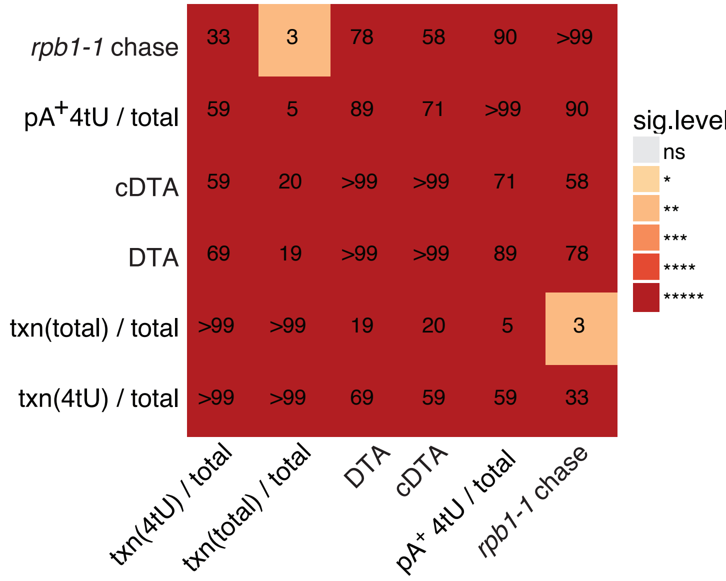

Figure S4, related to Figure 4

(A) Formulas to calculate DRs are shown on top. Bottom panels show theoretical accumulation of 4tU labeled RNA (yellow line) over time compared to steady-state total RNA (black dashed line) of 4 hypothetical genes with high (left panels) and low (right panels) transcription rates (k_{txn}) or high (top panels) or low (bottom panels) DRs (k_{decay}). Blue dotted line shows the amount of RNA transcribed since timepoint 0. Note that k_{txn} is represented by the slope of the blue dotted line.

(B) Significance of correlations shown in Figure 4C depicted as in Figure S2B.

sample_name (EPAP_fraction_strain_minrapamycin_replicate)	Raw		quality filtering	uniquely mapped reads mapped to S.cervisiae - S.pombe joint genome		Final S. cerevisiae reads after filtering genomic A-rich positions
	reads	bases	output reads	mapped to S. cerevisiae	mapped to S. Pombe	
noPap_Nab2AA_input_0_1	3334641	166732050	3282063	2595050	42095	2313940
noPap_Mex67AA_input_0_1	3054292	152714600	3002568	2467734	41709	2202503
noPap_Nab2AA_ip_0_1	3056716	152835800	2929431	2015728	377298	1769572
noPap_Mex67AA_ip_neg0_1	5253477	262673850	5060704	3517377	795545	3116325
noPap_Mex67AA_ip_0_1	4038165	201908250	3883937	2949235	456822	2579454
xPap_Nab2AA_input_0_1	5162819	258140950	4787399	3944905	40380	3822077
xPap_Mex67AA_input_0_1	4916981	245849050	4442576	3781260	35565	3654903
xPap_Nab2AA_ip_0_1	3933492	196674600	3394680	2057164	434311	1888025
xPap_Nab2AA_ip_0_2	4866432	243321600	4448755	3040507	192401	2815418
xPap_Nab2AA_ip_0_3	4189502	209475100	3675178	2446311	163220	2214857
xPap_Mex67AA_ip_neg0_1	5649907	282495350	5045569	3579615	435360	3153307
xPap_Mex67AA_ip_0_1	4690443	234522150	4153541	2855569	151361	2648241
xPap_Mex67AA_ip_0_2	5035947	251797350	4645361	3156133	175134	3022976
xPap_Mex67AA_ip_0_3	3847037	192351850	3658720	2459241	132147	2310471

Table S1, related to Figure 1
Overview of RNA-seq libraries.
